# Distant Influence of Kuroshio Eddies on North Pacific Weather Patterns?

**DOI:** 10.1038/srep17785

**Published:** 2015-12-04

**Authors:** Xiaohui Ma, Ping Chang, R. Saravanan, Raffaele Montuoro, Jen-Shan Hsieh, Dexing Wu, Xiaopei Lin, Lixin Wu, Zhao Jing

**Affiliations:** 1 Department of Oceanography, Texas A&M University, College Station, TX, USA; 2 Physical Oceanography Laboratory/Qingdao Collaborative Innovation Center of Marine Science and Technology, Ocean University of China, Qingdao, PRC; 3 Department of Atmospheric Sciences, Texas A&M University, College Station, TX, USA

## Abstract

High-resolution satellite measurements of surface winds and sea-surface temperature (SST) reveal strong coupling between meso-scale ocean eddies and near-surface atmospheric flow over eddy-rich oceanic regions, such as the Kuroshio and Gulf Stream, highlighting the importance of meso-scale oceanic features in forcing the atmospheric planetary boundary layer (PBL). Here, we present high-resolution regional climate modeling results, supported by observational analyses, demonstrating that meso-scale SST variability, largely confined in the Kuroshio-Oyashio confluence region (KOCR), can further exert a significant distant influence on winter rainfall variability along the U.S. Northern Pacific coast. The presence of meso-scale SST anomalies enhances the diabatic conversion of latent heat energy to transient eddy energy, intensifying winter cyclogenesis via moist baroclinic instability, which in turn leads to an equivalent barotropic downstream anticyclone anomaly with reduced rainfall. The finding points to the potential of improving forecasts of extratropical winter cyclones and storm systems and projections of their response to future climate change, which are known to have major social and economic impacts, by improving the representation of ocean eddy–atmosphere interaction in forecast and climate models.

Midlatitude oceanic fronts, such as the Kuroshio and Gulf Stream extension, play an anchoring role for atmospheric storm tracks through their effect on low-level baroclinicity in the troposphere^1^,^2^. However, the extent to which the variability of these oceanic fronts can have an impact on atmospheric storm tracks and weather patterns has been a topic of vigorous debate. Some early studies indicate that midlatitude SST-forced atmospheric response is generally weak compared to variability generated by atmospheric internal dynamics, casting doubt on the effectiveness of large-scale midlatitude SST anomalies forcing the atmosphere^3^. More recent studies focus on the effect of strong SST gradients along oceanic fronts on the atmosphere^4^,^5^,^6^,^7^, finding that SST anomalies associated with shifts of the oceanic fronts may cause changes in atmospheric storm tracks^8^,^9^,^10^,^11^. Emerging research has also begun to explore the role of ocean eddies along the fronts in affecting local rainfall and other weather variables, revealing a significant increase of rainfall paired with warm meso-scale ocean eddies^12^ and a meso-scale wind convergence induced by warm eddies^13^. Few of the existing studies^14^,^15^, however, explicitly address the potential role of meso-scale SST variability associated with ocean eddies in remotely influencing atmospheric storm track and winter rainfall variability, even though it has been well established that energetic eddies along the oceanic fronts can generate strong meso-scale SST anomalies, varying on seasonal-to-decadal time scales^16^.

## Results

**Figure 1:**
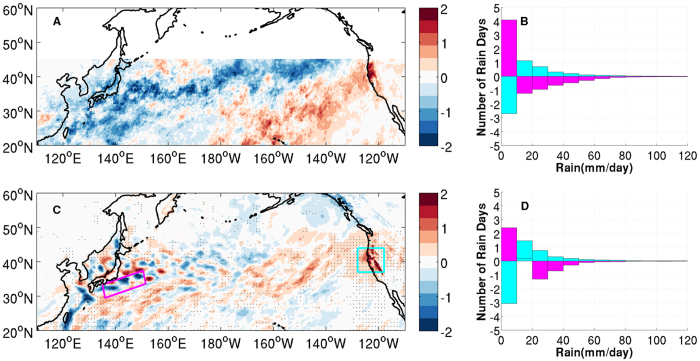
Observed and Simulated rainfall changes in response to meso-scale SST forcing. (**A**) Difference of TRMM winter season (NDJFM) mean rainfall (mmd^−1^) between Inactive Eddy Years (IEYs) and Active Eddy Years (AEY). (**B**) TRMM daily rainfall PDF difference between IEYs and AEYs along the Kuroshio (magenta) and over the U.S. North Pacific coast (cyan). (**C**) Difference of winter season (NDJFM) mean total rainfall (mmd^−1^) between two ensembles of 10 WRF simulations, MEFS (without ocean eddies) and CTRL (with ocean eddies). Rainfall difference significant at 95% confidence level based on a two-sided Wilcoxon rank sum test is shaded by gray dots. (**D**) Daily rainfall PDF difference between MEFS and CTRL along Kuroshio (magenta) and over the U.S. North Pacific coast (cyan). For both TRMM3B42 and simulated rainfiall, the PDF was derived by counting the number of rainy days in different rain rate ranges at each grid point and then averaging them in the region along the Kuroshio (denoted by the magenta box in (**C**)) and over the U.S. North Pacific coast (denoted by the cyan box in (**C))**. The maps were generated using M_Map V1.4 package for Matlab (http://www.eos.ubc.ca/~rich/map.html).

There is thought-provoking observational evidence pointing to a relationship between Kuroshio meso-scale SST variability and winter precipitation along the U.S. Northern Pacific coast. Figure 1A shows the difference of winter season mean rainfall composite, along with the corresponding daily rainfall probability density function (PDF) distribution difference, between five years [2002–2005,2010] when Kuroshio eddy activity was subdued and another five years [2000, 2006–2009] when the eddy activity was enhanced. The rainfall and ocean eddy statistics were derived from the high-resolution rainfall data from the Tropical Rainfall Measuring Mission (TRMM3B42)^17^ and sea-surface height (SSH) data from the TOPEX/Poseidon and other ocean surface topography measurement missions^18^, respectively. The selection of the eddy active and inactive years follows a recent study^19^ that identified these years as Kuroshio unstable and stable periods. It is evident that a weak Kuroshio eddy activity corresponds to a reduced (enhanced) winter precipitation along the Kuroshio extension (the U.S. Northern Pacific coast), and vice versa. Along with the change in the winter mean precipitation, there is a marked difference in the daily rainfall PDF distribution with increased (decreased) occurrence of heavy rain events along the Kuroshio when eddies are active (inactive), but the opposite over the eastern North Pacific (Fig. 1B). This observed relationship between meso-scale SST and precipitation variability is intriguing, but its robustness and causality need further scrutiny, because it is based on a short record when the high-resolution TRMM and SSH data overlap. However, the lack of long records of high-resolution observations capable of resolving ocean meso-scale eddies and their impact on the atmosphere makes it challenging to observationally validate the finding. For this reason, we turn to high-resolution regional climate model simulations.

We designed a set of twin numerical experiments using the Weather Research and Forecasting (WRF) model with 27 km horizontal resolution configured for the entire North Pacific sector. Each of the twin experiments consists of an ensemble of 10 simulations forced with MicroWave InfradRed Optimal Interpolated (MW-IR) daily SST at 0.09° spatial resolution from October 1, 2007 to March 31, 2008, a period that was marked by strong eddy activity but with near neutral phase of El Niño-Southern Oscillation (ENSO) and of Pacific Decadal Oscillation (PDO) (*Methods*). The only difference between the twin experiments is that in control simulations (CTRL) the MW-IR SST was used, whereas in meso-scale eddy filtered simulations (MEFS) lowpass filtered MW-IR SST with a 2-D spatial Loess filter^20^ of a 15° × 5° cut-off wavelength was used to remove meso-scale SST variability (Fig. S1). As such, the difference in atmospheric response between the two experiments can be attributed to meso-scale SST variability, allowing us to assess the impact of ocean eddies on winter-season storm track variability over the North Pacific. We note that, given the same ensemble size, the regional modeling approach improves signal-to-noise ratio over global modeling approach because identical lateral boundary conditions were used for all the ensemble members. The sensitivity of the results to different filter scales is not discussed here and needs to be further investigated.

**Figure 2:**
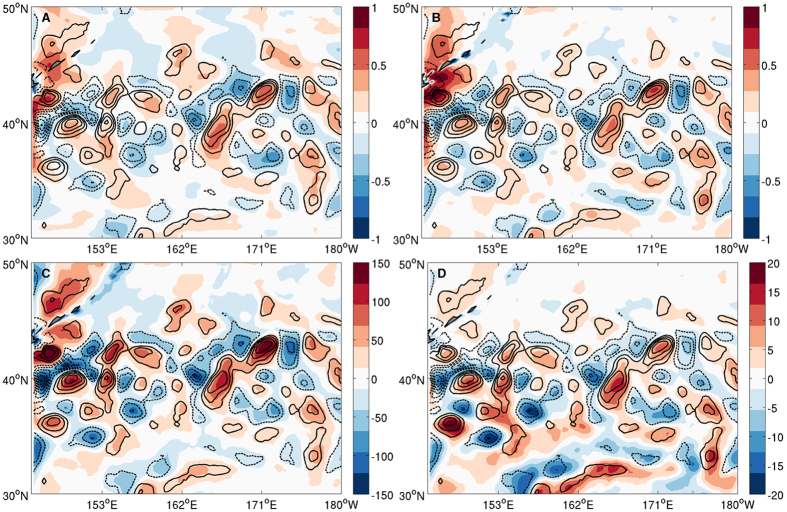
Satellite observed and model simulated frontal- and meso-scale air-sea interactions in KOCR. (**A**) 2007/8 winter season mean (NDJFM) spatially highpass-filtered SST (contour with interval of 0.5 °C) and 10 m wind speed (color in ms^−1^) derived from MW-IR and CCMP satellite observations (**B**) and from the ensemble mean of 27 km uncoupled WRF CTRL simulations, (**C**) highpass-filtered SST (contour with interval of 0.5 °C) and PBL (color in m), (**D**) highpass-filtered SST (contour with interval of 0.5 °C) and CAPE (color in Jkg^−1^) derived from the ensemble mean of 27 km uncoupled WRF CTRL simulations. The maps were generated using M_Map V1.4 package for Matlab (http://www.eos.ubc.ca/~rich/map.html).

Previous analyses of high-resolution satellite observations show that meso-scale SST anomalies can force a well-defined local response in near-surface atmospheric fields^21^,^22^,^23^. Figure 2A reveals a striking correspondence between the winter-mean meso-scale SST and surface winds derived from high-resolution satellite data in the KOCR during 2007/8 winter. This observed meso-scale SST and surface wind relationship is reproduced (Fig. 2B) with remarkably high fidelity in CTRL, giving the confidence that WRF is capable of faithfully representing frontal and meso-scale air-sea interactions in the KOCR. Furthermore, the model simulates a highly cohesive response to the meso-scale SST forcing in atmospheric PBL height (Fig. 2C) and convective available potential energy (CAPE) (Fig. 2D), as well as convective rainfall and air-sea turbulent heat fluxes (not shown), suggesting that the influence of the meso-scale SST extends throughout the entire PBL and beyond. A natural question arises from these results: Can these Kuroshio eddies have far-reaching impacts on the Pacific storm track and weather patterns further downstream?

A comparison of simulated winter precipitation between CTRL and MEFS clearly shows that in the absence of meso-scale SST (i.e., MEFS), the simulated winter-mean rainfall exhibits not only a significant decrease along the upper branch of the Kuroshio, but also enhanced winter precipitation over the eastern North Pacific, particularly along the U.S. Northern Pacific coast (Fig. 1C). In fact, the simulated precipitation difference pattern between MEFS and CTRL bears a discernible resemblance to the observed precipitation difference between the Kuroshio stable and unstable period (Fig. 1A). Furthermore, the PDFs of simulated daily rainfall display observationally consistent changes with increased (decreased) occurrence of daily heavy rain events along the Kuroshio (the U.S. Northern Pacific coast) in the presence of the meso-scale SST (Fig. 1D). The decrease in winter rainfall caused by the absence of oceanic eddies in MEFS amounts to 20–25% of the total winter mean rainfall in CTRL, which is comparable to the seasonal rainfall variation in this region. A further separation of the simulated rainfall into convective and large-scale (non-convective) rain demonstrates that the rainfall decrease along the Kuroshio is primarily caused by changes in convective rainfall, while the rainfall increase over the eastern basin is largely due to non-convective rainfall changes (Fig. S2). Orographic effects may contribute to the dramatic change of non-convective rainfall along the west coast of US.

**Figure 3:**
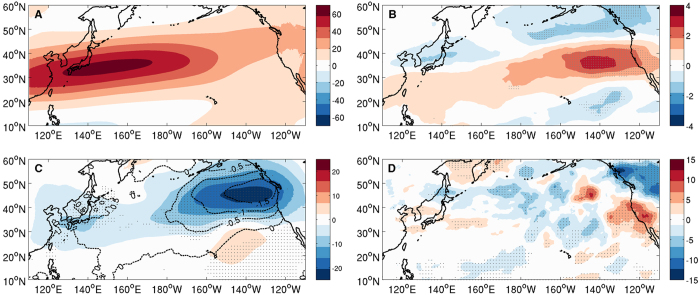
Upper atmospheric response to meso-scale SST forcing. (**A**) Winter season (NDJFM) mean zonal wind U at 300 hpa, U300 (ms^−1^), in CTRL. (**B**) Difference of U300 (ms^−1^), (**C**) Sea-Level-Pressure (mb) (contour) and geo-potential height at 500 hpa (Z500) (m) (color) and (**D**) transient eddy kinetic energy at 300 hpa (m^2^s^−2^) between MEFS and CTRL (MEFS-CTRL). The transient kinetic energy was derived using 2–8 day bandpass-filtered variables. In (**B**–**D**), the difference significant at 95% confidence level based on a two-sided Wilcoxon rank sum test is shaded by gray dots. The maps were generated using M_Map V1.4 package for Matlab (http://www.eos.ubc.ca/~rich/map.html).

Analyses of changes in upper atmospheric circulation between the twin experiments reveal a consistent jet stream and storm track response to meso-scale SST forcing. In MEFS where meso-scale SST variability is removed, the simulated jet stream in the upper atmosphere shifts equatorward in the eastern basin compared to CTRL (Fig. 3A,B). This shift is a part of an anomalous equivalent barotropic circulation that develops in the region (Fig. 3C) and corresponds to a dipole-like response in upper level storm track activity with an increase (decrease) storm activity to the south (north) of the jet stream (Fig. 3D). A similar equivalent barotropic response was also noted in a recent high-resolution modeling study that highlights the importance of high frequency SST forcing in driving the large-scale atmospheric response^15^. The region of enhanced storm track activity is co-located with the area of enhanced winter precipitation along the U.S. Northern Pacific coast, suggesting a close linkage between them. Collectively, these modeling results are supportive of the observational findings pointing to a role of Kuroshio eddies in remotely influencing Pacific storm track and winter precipitation variability.

**Figure 4:**
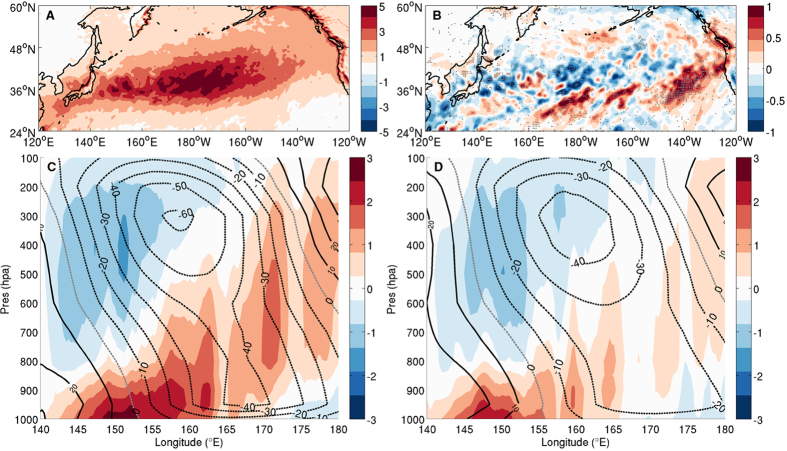
Simulated diabatic heating difference and synoptic baroclinic waves over the Kuroshio extension region during cyclone development periods in MEFS and CTRL. (**A**) Vertically integrated (from 1000 to 300 hpa) storm-day diabatic heating (Pa•K/s) in CTRL. (**B**) Difference of vertically integrated (from 1000 to 300 hpa) storm-day diabatic heating (Pa•K/s) (MEFS-CTRL). The difference significant at 95% confidence level based on a two-sided Wilcoxon rank sum test is shaded by gray dots. (**C**) Vertical structure of geopotential height (contours, m) and diabatic heating (shaded, Pa•K/s) along the storm path (denoted by yellow dotted lines in Fig. S4) for all the developing synoptic (2–8 day) storms in CTRL, composited during the simulated storm days (see *SI text* and Fig. S5 for details). (**D**) Same as (**C**) but for MEFS. The storm days were defined based on a 20-percentile higher threshold criteria using a surface turbulent heat flux (THF) index derived by averaging simulated daily THF over a 30°x10° area [140°E–170°E, 32°N–42°N] in the respective simulations (*SI text*). The maps were generated using M_Map V1.4 package for Matlab (http://www.eos.ubc.ca/~rich/map.html).

The prevailing view of storm track dynamics draws a link between storm track variability and changes in atmospheric baroclinicity driven by the underlying SST gradient^1^,^2^. However, removing meso-scale SST in MEFS yields little changes in the mean SST gradient over the Kuroshio extension, and thus has insignificant impact on the atmospheric baroclinicity, which is confirmed by comparing the Eady maximum growth rate^24^ between the twin experiments (Fig. S3). Probing deeper into the simulations reveals that meso-scale SST has instead a significant impact on the lower atmospheric moisture budget and diabatic heating, both of which are reduced over the KOCR when meso-scale SST is removed. A further division of the diabatic heating into two categories – one during cyclone periods (hereafter storm days) and the other during nonstorm days – shows that the reduction is most significant during storm days (Fig. 4), indicating an effect of meso-scale SST on cyclogenesis through modifying atmospheric moisture and diabatic processes. Indeed, a composite analysis of synoptic (2–8 day) storms over the cyclogenesis region shows that baroclinic wave amplitude in CTRL is about 25% stronger than that in MEFS and the associated diabatic heating in CTRL has double the strength of that in MEFS (Fig. 4C,D) (additional discussion in *SI text* and Figs S4 and S5). These results point to a rectified effect of meso-scale SST on atmospheric moisture and diabatic heating, which partly arises from the nonlinearity in the Clausius-Clapeyron saturation vapor pressure relationship that gives a disproportionately larger impact on boundary layer humidity from warm ocean eddies than cold eddies.

The argument for the moisture effect as being a prime cause of the different cyclone development between the two experiments is further corroborated by a transient eddy energetic analysis. When meso-scale SST variability is suppressed (i.e., MEFS), the diabatic energy conversion that regulates transient eddy energy gain from diabatic processes is decreased by nearly 45% over the KOCR during storm days. In contrast, the baroclinic energy conversion that regulates the eddy energy gain from mean available potential energy is barely reduced (<3%). Among all the diabatic processes, reduction in latent heating is most significant, which alone contributes to nearly 70% of the total reduction in eddy kinetic energy gain from eddy available potential energy, confirming the importance of the meso-scale SST induced diabatic effect on cyclogenesis (*SI text* and Fig. S6).

The modification in baroclinic wave characteristics by meso-scale SST in the KOCR can affect downstream development of storm events (Fig. S4), raising the possibility that the anomalous circulation shown in Fig. 3 can be largely attributed to an accumulated effect of the altered storm development in the eastern basin. Following a recent study^25^, we examine this possibility by accumulating the contribution of the extreme storm events to sea-level-pressure and geo-potential height in CTRL and MEFS. The resultant difference patterns in these fields between the two experiments bear a noteworthy resemblance to the equivalent barotropic circulation anomaly shown in Fig. 3C (*SI text* and Fig. S7), suggesting that the distant influence of Kuroshio eddies on the North Pacific weather pattern may indeed be understood in large measure as the cumulative effect of the altered extreme storm behavior due to changes in mesoscale SST forcing. We note that the E-vector divergence exhibits a consistent southward shift with the mean flow in the far eastern North Pacific (not shown), suggesting that transient eddy feedback onto the mean flow may also play a role in the southward shift of the jet stream in the region as depicted by the classic eddy-mean flow interaction theory.

## Discussion

The potential role of ocean eddies in modulating storm tracks and weather patterns has important implications for improving extratropical cyclone forecasts and seasonal climate prediction in midlatitudes, as well as for improving projections of the storm track response to future climate change. An auto-correlation analysis of the monthly mean standard deviation of satellite-derived meso-scale SSH variability in the KOCR indicates that the meso-scale ocean eddies can persist for up to 9 months in this region (Fig. S8), suggesting a significant predictability of meso-scale ocean eddy energetics at seasonal time scales. Through ocean eddy feedback onto the atmosphere, the persistence of the ocean system may provide an important source of predictability for the midlatitude climate system at seasonal time scales. Therefore, to improve forecast skills of mid-latitude winter storms, it may be imperative that dynamical seasonal prediction properly resolves meso-scale ocean eddies and the associated frontal-scale air-sea interactions. Furthermore, a recent study reveals that the eddy-rich western boundary current regimes have experienced the most rapid changes in the global ocean^26^, prompting the question of whether these changes in the boundary current systems and associated ocean eddies are important in our understanding of future storm track changes and whether climate models need to improve their representation of ocean-eddy atmosphere feedbacks in order to accurately project the storm track response to future climate change.

## Methods

We used the Weather Research and Forecasting (WRF) Model developed by National Center for Atmospheric Research (NCAR). WRF is designed to serve both weather forecasting and atmospheric research needs and has been used in variety of regional climate studies^27^. The computational domain covers the entire North Pacific Ocean from 3.6°N to 66°N, 99°E to 270°E. The horizontal grid is set at 27 km based on a Mercator projection. The model atmosphere is divided into 30 vertical levels. The model configuration includes Lin *et al.*’s scheme for microphysics^28^, RRTMG and Goddard scheme for longwave and shortwave radiation^29^,^30^, a Noah land surface scheme, YSU scheme for planetary boundary layer^31^, and a horizontal, first-order closure Smagorinsky scheme^32^ for calculating eddy coefficient. The cumulus parameterization used in the simulations is the Kain-Fritsch (KF) scheme^33^. The 6-hour low-boundary SST forcing from October 1, 2007 to March 31, 2008 was interpolated from the satellite derived MW-IR daily SST at 0.09° resolution. The initial condition and lateral boundary conditions for the same period were derived from the 6-hour NCEP/DOE AMIP-II Reanalysis (NCEP2) reanalysis data.

Two ensembles of WRF experiments were carried out to specifically test the effect of meso-scale SST variability associated with ocean eddies on the atmosphere. The two ensembles of runs, each of which has 10 members, only differ in the SST forcing field. The first ensemble of runs, which is referred to as the control experiment (CTRL), was forced with the 0.09° MW-IR SST, while the second ensemble, which is referred to as the meso-scale eddy filtered simulation (MEFS), was forced with a spatially lowpass-filtered SST to remove meso-scale eddies. Following previous studies^20^,^21^, we used a Loess filter^23^ with a 15° (longitude) × 5° (latitude) cut-off wavelength to remove meso-scale SST variability under approximate 800 km. A snapshot of the MW-IR and lowpass-filtered SST forcing used in each of the two simulations is shown in Fig. S1A,B, respectively. In each ensemble, the initial conditions for the 10 members were obtained from the NCEP2 reanalysis on the same day but in different years, i.e. October 1, 2002, 2003, 2004, 2005, 2006, 2007, 2008, 2009, 2010, 2011, respectively. All simulations were integrated for 6 months during the boreal winter season from October 1, 2007 to March 31, 2008 (ONDJFM), with the first month being omitted from the analyses to account for model spin-up. It is worth emphasizing that in this regional modeling approach the lateral boundary conditions are identical for the twin experiments, which improves the signal-to-noise ratio when compared to similar global model experiments. Therefore, an ensemble size of 10 is sufficient to detect the changes in storm activity with high statistical confidence.

## Additional Information

**How to cite this article**: Ma, X. *et al.* Distant Influence of Kuroshio Eddies on North Pacific Weather Patterns? *Sci. Rep.* **5**, 17785; doi: 10.1038/srep17785 (2015).

## Supplementary Material

Supplementary Information
