# Supplementary material for: Distant Influence of Kuroshio Eddies on North Pacific Weather Patterns?
Source: Sci Rep. 2015 Dec 4;5:17785. doi: 10.1038/srep17785 (PMC4669523; doi:10.1038/srep17785)
Supplement: Supplementary Information [file srep17785-s1.pdf]

Supplementary Information For

Distant Influence of Kuroshio Eddies on North Pacific Weather Patterns

Xiaohui Ma[1,2], Ping Chang[1,2,3,*], R. Saravanan[3], Raffaele Montuoro[3], Jen-Shan Hsieh[3], Dexing Wu[2], Xiaopei Lin[2], Lixin Wu[2], Zhao Jing[1,2]

1. Department of Oceanography, Texas A&M University, College Station, TX, USA
2. Physical Oceanography Laboratory/Qingdao Collaborative Innovation Center of Marine Science and Technology, Ocean University of China, Qingdao, PRC.
3. Department of Atmospheric Sciences, Texas A&M University, College Station, TX, USA

[*]Corresponding author. E-mail: ping@tamu.edu

Composite Analysis of Synoptic Storms

To examine the importance of meso-scale air-sea interaction in cyclogenesis, a composite analysis was performed for 2-8 day bandpass filtered atmospheric variables that characterize synoptic storm systems. The composites were based on simulated storm days selected using an 80-percentile threshold criteria for surface turbulent heat flux (THF) index over the KOCR. The THF index was derived by averaging simulated daily THF over a 30°x10° area [140°E-170°E, 32°N-42°N] in the respective simulations and a storm day is defined as one where the THF index exceeds 80 percentile value of the entire THF record[25,34]. The rationale behind using the THF index for the composite analysis is that THF is an effective measure of air-sea interaction and thus the THF-based composite can shed useful light on effects of air-sea interactions on storm development.

The THF criterion yields, on average, a total of 30 storm days in each 6-month WRF run, which occupies about 20% of total winter days from November to March. Fig. S4 shows the lag-composite of 2-8 day bandpass filtered meridional wind v at 850 hpa in CTRL (left) and MEFS (right) from lag -2 day to lag +2. Lag 0 corresponds to peak values of THF index when storms pass over the Kuroshio region. All the composited v fields were normalized by the maximum northerly v amplitude near the storm center at lag -3 day in CTRL and MEFS, respectively, so that the difference in storm evolution between CTRL and MEFS can be clearly visualized. The composite reveals a clear eastward propagating baroclinic wave pattern and it is evident that the wave intensifies more rapidly over the Kuroshio extension in CTRL than in MEFS. The difference in the wave amplitude growth between CTRL and MEFS is further illustrated in Fig. S5 that shows the maximum normalized northerly v as a function of lag. These results corroborate the finding shown in Fig. 4c&d of the main text that the synoptic baroclinic wave is about 25% stronger in CTRL than in MEFS, based on the storm-day composite of geopotential height along the storm path indicated by the yellow dotted line in Fig. S4c&d.

Transient Eddy Energetic Analysis

Define $T' = T - \overline{T}$ as transient eddy temperature perturbations from climatological temperature $\overline{T}$, the eddy temperature variance equation[35] can be used to diagnose eddy available potential energy (EAPE):

$$\frac{\partial}{\partial t}\overline{\left(\frac{T'^2}{2}\right)} = -\nabla\cdot\overline{\left(\frac{\boldsymbol{u}T'^2}{2}\right)} - \frac{\partial}{\partial z}\overline{\left(\frac{wT'^2}{2}\right)} - \overline{\boldsymbol{u}'T'}\cdot\nabla\overline{T} - \overline{w'T'}\frac{\partial\overline{T}}{\partial z} + \overline{T'\frac{\partial Q'}{\partial z}} \qquad (S1),$$

Eq. (S1) is formulated in z-coordinate, where Q is surface heat flux, $\boldsymbol{u}$ and w are the horizontal velocity vector and vertical velocity, the overbar represents time average and the prime represents perturbations. The left-hand-side (LHS) term of Eq. (S1) represents time rate of change of eddy temperature variance caused by horizontal and vertical advection of eddy temperature variance (1st and 2nd term on the right-hand-side (RHS)), horizontal and vertical eddy fluxes on mean temperature gradient (3rd and 4th term on RHS) and diabatic conversion (5th term on RHS). Q' comprises both radiative fluxes and non-radiative fluxes that includes boundary layer turbulent heat fluxes, convective fluxes and microphysics fluxes. An analysis of Q' shows that changes in the non-radiative fluxes dominate over changes in radiative fluxes when meso-scale SST is removed. Therefore, the results of diabatic conversion analysis shown in Fig. S6 include only the non-radiative heating in Q' . In computing the diabatic conversion, T' and Q' were derived first by removing the respective monthly mean climatology of a five-member ensemble mean (only five ensemble members save all necessary terms in the temperature equation to complete the budget analysis and the results are not sensitive to the definitions of climatology) and $T'\frac{\partial Q'}{\partial z}$ was then composited for storm days and nonstorm days, respectively. Fig. S6a&b show the winter season mean and storm-day diabatic conversion in CTRL, whereas Fig. S6c&d show the winter season mean and storm-day diabatic conversion difference between MEFS and CTRL (MEFS-CTRL). Clearly, the

storm-day diabatic conversion shows a significant reduction (~45%) along the Kuroshio extension [140°E-170°E, 32°N-42°N], suggesting that EAPE gain from diabatic energy source is significantly reduced during cyclogenesis.

It can be shown that the rate of change of eddy temperature variance described by Eq. (S1) is dominated by three major terms[36,37]:

$$\frac{\partial}{\partial t}\overline{\left(\frac{T'^2}{2}\right)} \approx -\overline{\boldsymbol{u}'T'}_D \cdot \nabla\bar{T} - \overline{w'T'}\frac{\partial \bar{T}}{\partial z} + \overline{T'\frac{\partial Q'}{\partial z}} \qquad (S2),$$

where 1st and 2nd term on RHS represent baroclinic conversion that regulates the eddy energy gain from mean available potential energy (MAPE) and conversion from EAPE to eddy kinetic energy (EKE)[37], respectively. We diagnosed Eq. (S2) by computing the eddy variance tendency (the term on LHS), EAPE-to-EKE conversion (2nd term on RHS) and diabatic conversion (3rd term on RHS) directly from model output, and estimating baroclinic conversion (1st term on RHS) indirectly as a residual. The results show that during storm days the baroclinic energy conversion is only slightly reduced (<3%) over the Kuroshio extension region [140°E-170°E, 32°N-42°N], in sharp contrast to a 45% reduction in the diabatic conversion when meso-scale SST is suppressed in MEFS. This is consistent with the Eady maximum growth analysis shown in Fig. S3. Since the tendency term is also close to zero, the reduction in the diabatic conversion is mostly balanced by a decrease in the EAPE-to-EKE conversion, resulting in weakened cyclogenesis over the Kuroshio extension region. Further analysis shows that among all

the diabatic processes, reduction in latent heating is most significant, which alone contributes to nearly 70% of the total reduction in the EAPE-to-EKE conversion.

Accumulated Storm Effect on Anomalous Equivalent Barotropic Circulation

A recent study shows that interannual-to-decadal variability in Aleutian Low sea-level pressure variability can be explained in large measure by an accumulated effect of extreme winter storms[25]. Here we examine the extent to which the equivalent barotropic circulation anomaly developed in the eastern North Pacific between CTRL and MEFS, as shown in Fig. 3, can be attributed to the difference in storm behavior between the two experiments. Following the methodology described in Ma et al[25], we first computed the contribution of extreme storm events to sea-level pressure (SLP) and geo-potential height at 500 hpa (Z500) by summing up the SLP and Z500 from the storm onset day to the subsequent 3 days, i.e., from lag 0 to lag 3 days in CTRL and dividing them by the count of all winter days. The definition of the storm onset days is the same as that described in the ***Composite Analysis of Synoptic Storms***, except that we used both the 70th and 80th THF criteria. The former yields a total number of storm days (from lag 0 to 3 days) of 39% of the entire winter days in the ensemble of 10 CTRL runs, while the latter yields 30%. We then repeated the same calculation for SLP and Z500 in MEFS using the same selected storm days. Fig. S7 shows the difference in SLP and Z500 between MEFS and CTRL (MEFS-CTRL) using both the THF criteria. It is evident that both difference patterns bears a noteworthy resemblance to the SLP and Z500 pattern in Fig. 3c. To quantify the similarity between these patterns, we carried out a pattern correlation

analysis, revealing that about 79% (94%) of variance in the anomalous SLP shown in Fig. 3c can be explained by the storm accumulated pattern in Fig. 7S when using the 70th (80th) THF criterion. These results strongly suggest that the equivalent barotropic circulation anomaly can be largely attributed to the accumulated storm effect. In summary, the remote atmospheric response in the eastern North Pacific arises due in large measure to the fact that the absence of mesoscale SST forcing in MEFS alters the downstream development of the extreme storm events in CTRL, which in turn has an accumulated effect on the large-scale atmospheric circulation.

Wilcoxon rank sum significance test

The significance test we used is a two-sided Wilcoxon rank sum test. The main reason for choosing this test is that rainfall distribution is non-gaussian and thus does not satisfy the assumptions of the student t-test. The Wilcoxon rank sum test is a non-parametric test and has no requirement on the distribution[38,39]. In applying this test, we first calculated the de-correlation timescale of the analyzed variables to compute the degrees of freedom of the time series. The e-folding autocorrelation timescale of 6-hourly rainfall anomalies in our experiments is less than one-day (~ about 9 hours), suggesting that daily mean rainfall time series provide independent data points. We then concatenated the daily mean data from all 10 ensemble members at each grid point (1500 independent data points) and computed the daily mean rainfall distribution for each of the experiments. Finally, the median values of the two distributions were tested to see if they are significantly different based on the Wilcoxon rank sum test.

References

34 Shaman, J., Samelson, R. M. & Skyllingstad, E. Air-sea fluxes over the Gulf Stream region: Atmospheric controls and trends. *J Climate* **23**, 2651-2670 (2010).

35 Garratt, J. R. *The atmospheric boundary layer*. (Cambridge University Press, 1992). 32.

36 Eden, C., Greatbatch, R. J. & Olbers, D. Interpreting eddy fluxes. *J Phys Oceanogr* **37**, 1282-1296, doi:Doi 10.1175/Jpo3050.1 (2007).

37 Marshall, J. & Shutts, G. A Note on Rotational and Divergent Eddy Fluxes. *J Phys Oceanogr* **11**, 1677-1680, doi:Doi 10.1175/1520-0485(1981)011<1677:Anorad>2.0.Co;2 (1981).

38 Mann, H. B. & Whitney, D. R. On a Test of Whether One of 2 Random Variables Is Stochastically Larger Than the Other. *Ann Math Stat* **18**, 50-60, doi:Doi 10.1214/Aoms/1177730491 (1947).

39 Fay, M. P. & Proschan, M. A. Wilcoxon-Mann-Whitney or t-test? On assumptions for hypothesis tests and multiple interpretations of decision rules. *Statist Surv* **4**, 1-39, doi:10.1214/09-SS051 (2010).

Figures in Supporting Information

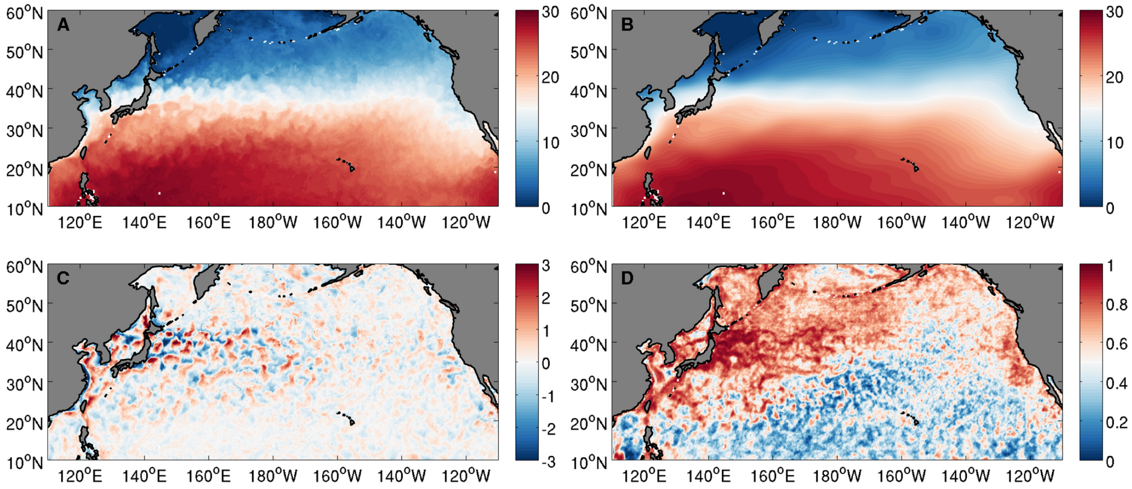

Figure S1*. *High-resolution MW-IR satellite derived SST and the corresponding spatially filtered SST. *A snapshot of the daily SST at 0.09° spatial resolution on January 1, 2008 (**A**) and the lowpass-filtered SST on the same day using the Loess filter (**B**). The difference between the two SSTs is shown in (**C**). All the SSTs are in °C. The 0.09° MW-IR SST (**A**) from October 1, 2007 to March 31, 2008 was used to force WRF in the CTRL run, while the lowpass-filtered SST (B) during the same period was used to force WRF in the MEFS run. The fraction of the MW-IR SST anomaly variance (seasonal cycle removed) explained by the meso-scale SST (**C**) during November 1, 2007 through March 31, 2008 is shown in (**D**). The maps were generated using M_Map V1.4 package for Matlab (http://www.eos.ubc.ca/~rich/map.html).*

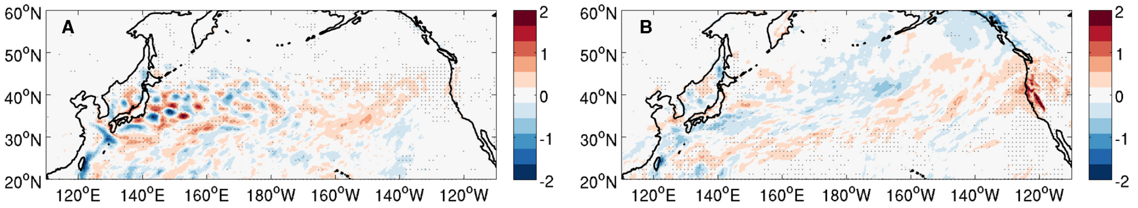

Figure S2. Simulated convective and non-convective rainfall differences between MEFS and CTRL*. Convective (****A****) and non-convective (****B****) winter (NDJFM) season mean rainfall difference (mmd^{-1}) between the two ensembles of 10 WRF simulations, MEFS and CTRL. Rainfall difference significant at 95% confidence level based on a two-sided Wilcoxon rank sum test is shaded by gray dots. The maps were generated using M_Map V1.4 package for Matlab (http://www.eos.ubc.ca/~rich/map.html).*

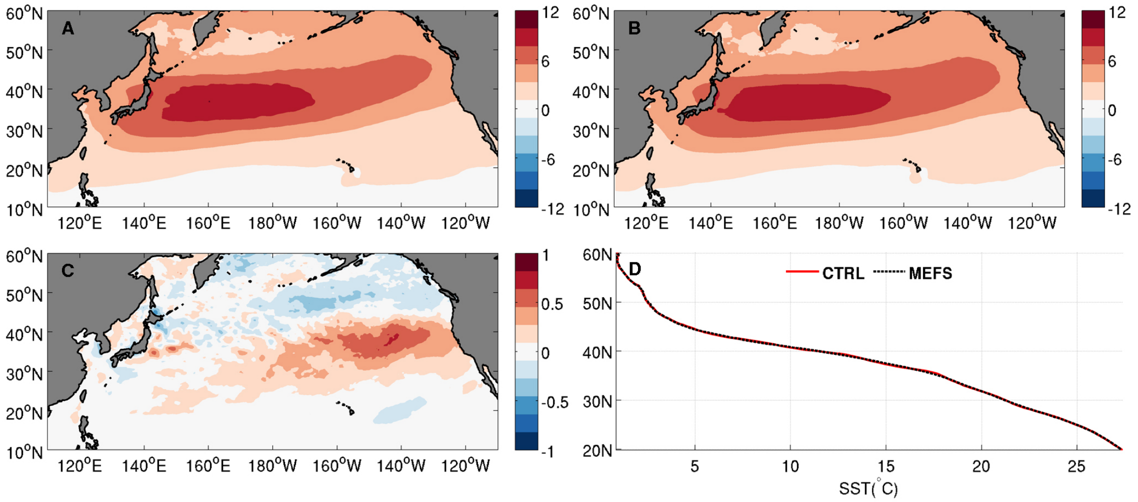

Figure S3. Atmospheric baroclinicity and zonally averaged SST over the Kuroshio extension. *Winter mean Eady growth rate at 850 hpa in CTRL (****A****) and MEFS (****B****), respectively. Eady growth rate difference between MEFS and CTRL (MEFS-CTRL) (****C****). Zonally averaged MW-IR (red) and lowpass filtered (black) SST between 140°E and 180°E (****D****). The maps were generated using M_Map V1.4 package for Matlab (http://www.eos.ubc.ca/~rich/map.html).*

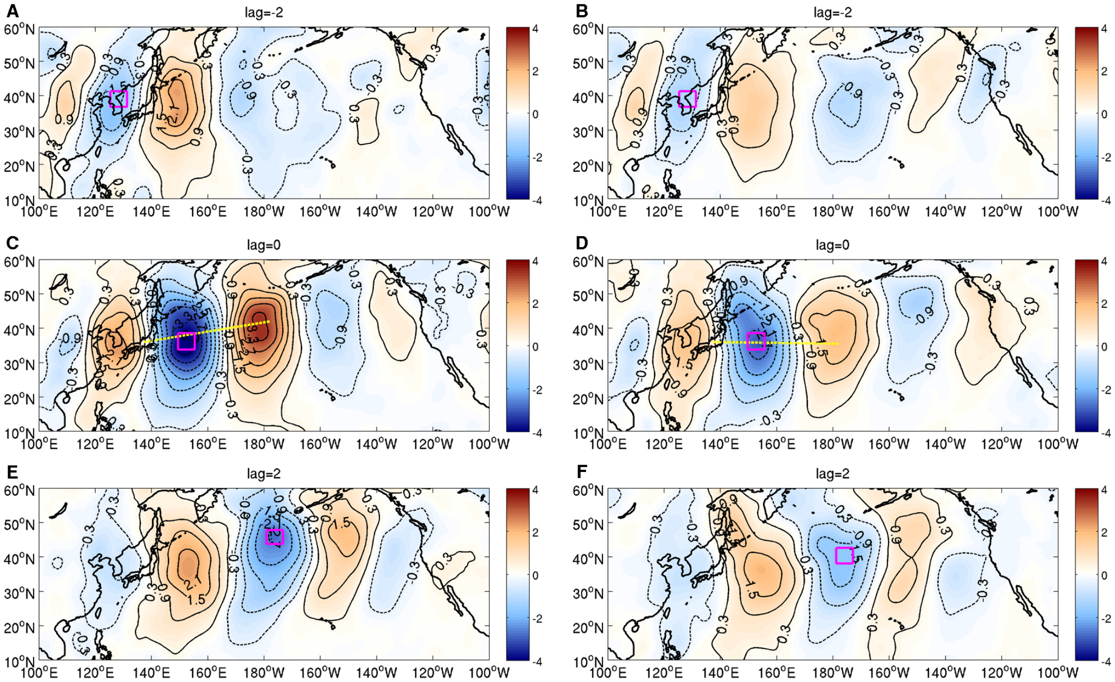

Figure S4. Simulated synoptic storm development. *Lag-composite of 2-8 day bandpass filtered meridional wind v at 850 hpa at lag -2, 0, 2 days for CTRL (A,C,E) and MEFS (B,D,F), respectively. In each case, the composites are normalized by the maximum v at lag -3 days at which the storm center is about to enter the ocean from the Eurasian continent. The dotted-lines in lag 0 composites indicate the storm path along which the vertical structure of the composited storm is shown in Fig. 4C&D. The square in each panel defines a 5°X5° square following* $Max(|v'|)$*. The* $Max(|v'|)$ *value averaged over the square area is used to compute storm amplitude evolution in FigureS5. The maps were generated using M_Map V1.4 package for Matlab (http://www.eos.ubc.ca/~rich/map.html).*

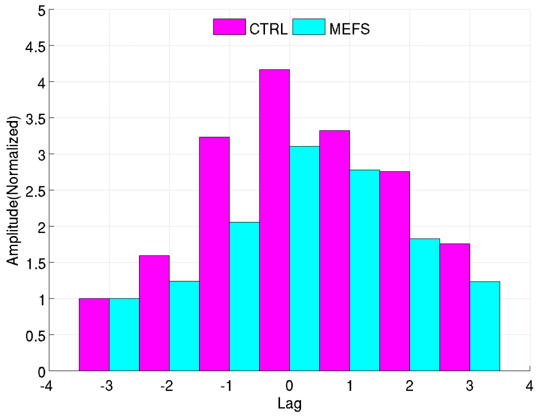

Figure S5. Composited synoptic wave amplitude evolution. *Histograms of maximum normalized northerly v averaged over the square in each panel of Fig. S4 as a function of*

lag, which gives a measure of time evolution of the average storm intensity in CTRL (magenta) and MEFS (cyan).

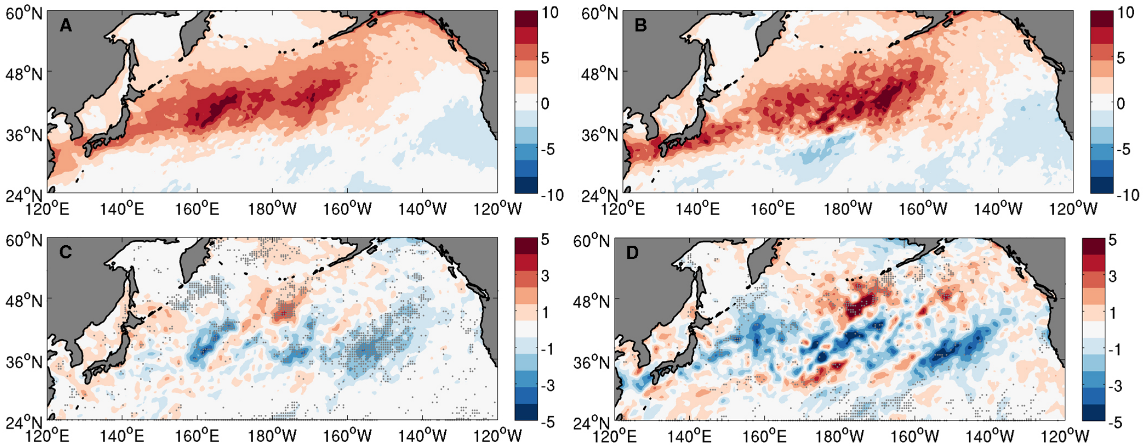

Figure S6. Diabatic conversion to transient eddy available potential energy. *Vertically integrated (from 1000 to 300 hpa) diabatic conversion,* $\overline{T'\frac{\partial Q'}{\partial z}}$*, where* T' *and* Q' *represent anomalies of atmospheric temperature and diabatic heating after removing seasonal cycle, averaged over the entire winter season (**A**) and contribution from storm days only (**B**) in CTRL. Diabatic conversion difference between MEFS and CTRL (MEFS-CTRL) during the entire winter season (**C**) and storm days only (**D**). Difference significant at 95% confidence level based on a two-sided Wilcoxon rank sum test is shaded by gray dots. The maps were generated using M_Map V1.4 package for Matlab (http://www.eos.ubc.ca/~rich/map.html).*

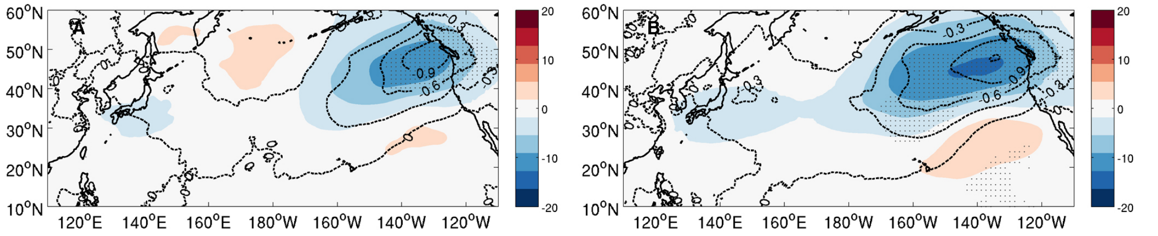

Figure S7. Accumulated effect of winter extreme storm events on anomalous equivalent barotropic circulation. *Difference in Sea-Level-Pressure (contour, mb) and geo-potential height at 500 hpa (Z500) (color, m) between MEFS and CTRL, computed by totaling the contribution during storm days in CTRL. The storm days were defined using 70*th *(**A**) and 80*th *(**B**) percentile THF threshold in CTRL and used for both CTRL and MEFS. Difference significant at 95% confidence level based on a two-sided Wilcoxon rank sum test is shaded by gray dots. The maps were generated using M_Map V1.4 package for Matlab (http://www.eos.ubc.ca/~rich/map.html).*

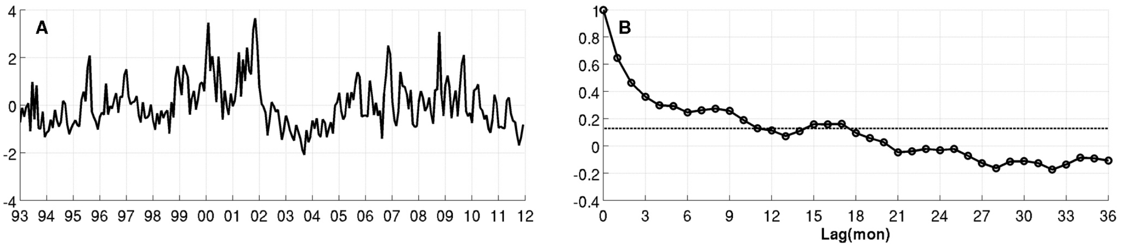

Figure S8. Time series of satellite observed SSH variability over the KOCR and its auto-correlation. *(**A**) Normalized time series of area-averaged quasi-monthly (4 weeks) variance anomalies of meso-scale SSH in the KOCR [142°E-150°E, 35°N-40°N]. (**B**) Auto-correlation of the time series shown in (**A**). The horizontal line indicates the upper confidence bound at 95% significant level based on a student t-test.*
